# 3D-Image-Guided Multi-Catheter Interstitial Brachytherapy for Bulky and High-Risk Stage IIB–IVB Cervical Cancer

**DOI:** 10.3390/cancers14051257

**Published:** 2022-02-28

**Authors:** Tetsuya Kokabu, Koji Masui, Yosuke Tarumi, Naoki Noguchi, Kohei Aoyama, Hisashi Kataoka, Hiroshi Matsushima, Kaori Yoriki, Daisuke Shimizu, Hideya Yamazaki, Kei Yamada, Taisuke Mori

**Affiliations:** 1 Department of Obstetrics and Gynecology, Kyoto Prefectural University of Medicine, Graduate School of Medical Science, Kyoto 602-8566, Japan; y-tarumi@koto.kpu-m.ac.jp (Y.T.); k-aoyama@koto.kpu-m.ac.jp (K.A.); hlm-11@koto.kpu-m.ac.jp (H.K.); m-hiro@koto.kpu-m.ac.jp (H.M.); kaorix26@koto.kpu-m.ac.jp (K.Y.); moriman@koto.kpu-m.ac.jp (T.M.); 2 Department of Radiology, Kyoto Prefectural University of Medicine, Graduate School of Medical Science, Kyoto 602-8566, Japan; mc0515kj@koto.kpu-m.ac.jp (K.M.); nguchi@koto.kpu-m.ac.jp (N.N.); dshimizu@koto.kpu-m.ac.jp (D.S.); yamahi@koto.kpu-m.ac.jp (H.Y.); kyamada@koto.kpu-m.ac.jp (K.Y.)

**Keywords:** cervical cancer, interstitial brachytherapy, ambulatory technique, CT, MRI

## Abstract

**Simple Summary:**

The prognosis of locally advanced cervical cancer still remains poor. Recently, image-guided brachytherapy ameliorated local control and pelvic control in these patients. Additionally, concurrent chemoradiotherapy with interstitial brachytherapy (ISBT) demonstrated more favorable outcomes than that with intracavity brachytherapy. The purpose of our study was to evaluate the efficacy and safety of CT-MRI-guided multi-catheter ISBT for bulky (≥4 cm) and high-risk stage IIB-IVB cervical cancer. Total of 18 patients with squamous cell carcinoma received concurrent chemoradiotherapy with ISBT were assessed. Four (22.2%), seven (38.9%), and seven (38.9%) patients were diagnosed with stage II, III, and IV cervical cancer, respectively. The four-year local control, pelvic control, disease-free survival, and overall survival rates were 100%, 100%, 81.6%, and 87.8%, respectively. Although three (16.7%) patients experienced grade 3 late adverse events, no one had procedure-related complications. CT-MRI-guided multi-catheter ISBT could be a promising treatment strategy for locally advanced cervical cancer.

**Abstract:**

This study aimed to evaluate the efficacy and safety of computed tomography-magnetic resonance imaging (CT-MRI)-guided multi-catheter interstitial brachytherapy for patients with bulky (≥4 cm) and high-risk, stage IIB–IVB advanced cervical cancer. Eighteen patients who underwent concurrent chemoradiotherapy with multi-catheter interstitial brachytherapy between September 2014 and August 2020 were enrolled. The prescribed dose of external beam radiotherapy was 45–50.4 Gy, and the brachytherapy high-dose-rate aim was 25–30 Gy per 5 fractions. The endpoints were four-year local and pelvic control rates, four-year disease-free and overall survival rates, and the adverse events rate. The median follow-up period was 48.4 months (9.1–87.5 months). Fifteen patients received concurrent cisplatin therapy (40 mg/m^2^, q1week). Four (22.2%), seven (38.9%), and seven (38.9%) patients had stage II, III, and IV cervical cancer, respectively. Pelvic and para-aortic lymph node metastases were observed in 11 (61.1%) and 2 (11.1%) patients, respectively. The median pre-treatment volume was 87.5 cm^3^. The four-year local control, pelvic control, disease-free survival, and overall survival rates were 100%, 100%, 81.6%, and 87.8%, respectively. Three (16.7%) patients experienced grade 3 adverse events, and none experienced grade 4–5 adverse events. CT-MRI-guided multi-catheter interstitial brachytherapy could be a promising treatment strategy for locally advanced cervical cancer.

## 1. Introduction

Uterine cervical cancer is the fourth most common malignancy among women, with 570,000 new cases and 311,000 deaths per year worldwide [1]. While patients with distant metastasis or recurrence demonstrate poor prognosis, those with locally advanced cancer can generally be cured due to their sensitivity to radiation therapy. The standard definitive radiation therapy modalities for cervical cancer include external beam radiotherapy (EBRT) and intracavity brachytherapy (ICBT) with concurrent chemotherapy. The JGOG1066 study, a phase II trial from Japan of concurrent chemoradiotherapy (CCRT) with high-dose-rate ICBT, revealed that the 2-year local control (LC) and pelvic control (PC) rates in patients with cervical cancer <5 cm in diameter were 85% and 73.2%, respectively [2]. Moreover, the results indicated that bulky tumors >5 cm in diameter were linked to a lower LC rate, leading to poor prognosis [2]. In recent years, the technology of 3-dimensional (3D)-image-guided brachytherapy (IGBT) with computed tomography (CT) or magnetic resonance imaging (MRI) has been revolutionized, and the therapeutic effects of IGBT on cervical cancer have been confirmed in several mono-institutional reports [3,4]. In addition, the Groupe European de Curietherapie-European Society for Radiotherapy and Oncology (GEC-ESTRO) GYN Working Group, established to support and shape the emerging field of IGBT, has designed and initiated multicenter studies on this treatment modality [5,6]. CCRT with IGBT has led to long-term LC in patients with all stages of locally advanced cervical cancer [5,7]. On the other hand, dose-volume analysis revealed that cases with large or asymmetrical tumors occasionally did not receive a sufficient dose prescription, leading to a comparatively low LC rate. Consequently, the 5-year relative survival rate for patients with stage I cervical cancer was 92%; however, this number decreased to 57% for more advanced cervical cancer that has spread beyond the cervix and uterus to regional lymph nodes [8]. Therefore, the development of new therapeutic strategies for bulky cervical cancer is urgently required. 

Recently, it was reported that interstitial brachytherapy (ISBT) combined with ICBT enabled the coverage of the lesion to which ICBT alone was insufficient to prescribe adequate dose [4]. In particular, a combined ICBT and ISBT was a better choice for the patients with tumor larger than 4 cm in diameter [9]. However, some patients with cervical cancer are unsuitable for ICBT; thus, ISBT may be an indispensable clinical approach to treat these patients. Multi-catheter ISBT not only increases flexibility in dose distribution, but also reduces the dose to organs at risk (OARs). However, there are a few institutions that treat locally advanced cervical cancer with classical ISBT using multiple catheters without ICBT, because the insertion of catheters needs a meticulous technique. Therefore, no previous reports showed over three-year clinical outcomes with image-guided ISBT for locally advanced cervical cancer. Thus, since 2014, we have adopted the use of 3D-image-guided multi-catheter ISBT without ICBT for patients with cervical cancer with bulky (≥4 cm), extended tumors, or with vaginal stenosis in which ICBT applicators could not be inserted. Herein, we report the procedure and four-year clinical outcomes of multi-catheter ISBT for patients with bulky and high-risk locally advanced cervical cancer (FIGO 2008 stage IIB with node-positive or stage III-IVA) at our institution.

## 2. Materials and Methods

### 2.1. Patient Selection and Characteristics

**Figure 1:**
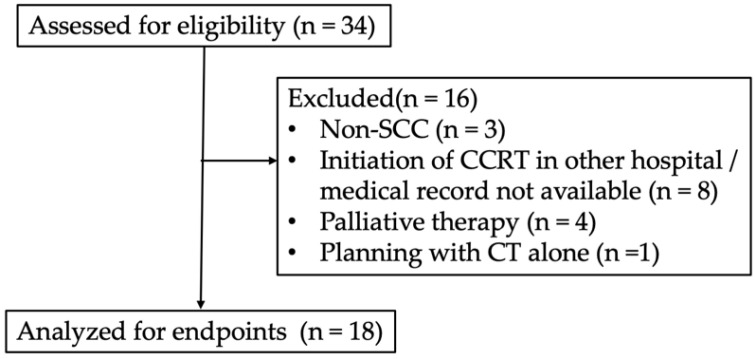
Flow diagram of participants with detailed information on excluded patients.

This retrospective study was approved by the institutional review board of Kyoto Prefectural University of Medicine (ERB-C-1403). A total of 34 patients with cervical cancer who underwent IGBT at our institution between September 2014 and August 2020 were included (Figure 1). Three patients had non-squamous cell carcinoma (SCC) cervical cancer. Four patients, including one with osseous metastasis, two with peritoneal dissemination, and one aged 95 years, treated as palliative therapy, and one with no records of MRI during planning were excluded. Since eight patients were referred to our hospital during CCRT or returned to other hospitals after ISBT, their medical records were not available. Finally, 18 patients were included in the analysis. Clinical disease staging was classified according to the International Federation of Gynecology and Obstetrics 2008. Clinical and pathological characteristics are summarized in Table 1. The median age was 64.5 (range: 24–82) years. The median follow-up duration for all patients was 48.4 (range: 9.1–87.5) months. All patients were pathologically diagnosed with squamous cell carcinoma >40 mm in diameter; four (22.2%) patients had stage IIB, seven (38.9%) had stage IIIB, five (27.8%) had stage IVA, and two (11.1%) had stage IVB cervical cancer. Furthermore, 11 (61.1%) patients demonstrated lymph node swelling detected by MRI and 18F-fluorodeoxyglucose-positron emission tomography/CT.

### 2.2. Definitive Radiation Therapy for Cervical Cancer

In accordance with the National Comprehensive Cancer Network guidelines, CCRT was performed for patients with stage IIB, III, IVA, and IVB with only para-aortic lymph node metastasis. Although weekly cisplatin (CDDP, 40 mg/m^2^) was recommended during CCRT [2], weekly CDDP was omitted for patients with renal failure or those older than 75 years of age. Definitive radiation therapy modalities include combined EBRT and high-dose-rate (HDR) brachytherapy. In this study, EBRT was delivered at a dose of 1.8–2 Gy per fraction five times a week until the total dose reached 50–50.4 Gy. Central shielding (CS) following whole pelvic EBRT (WPRT) was performed to reduce the dose to the rectum and bladder. CS was inserted at a total dose of 30 Gy for patients with stage IIB and III cervical cancer and 39.6–40 Gy for patients with stage IVA cervical cancer. WPRT was delivered using the 4-field box technique in 3D conformal radiotherapy, and CS was delivered using parallel opposing fields. An additional boost dose of 6 Gy was administered to patients with pelvic lymph node metastasis. In patients with stage IVB, EBRT was extended to the para-aortic lymph node at a total dose of 45 Gy, followed by a 10 Gy boost to the metastatic lesion in the lymph node. EBRT was omitted on days of brachytherapy administration.

### 2.3. Interstitial Brachytherapy

**Figure 2:**
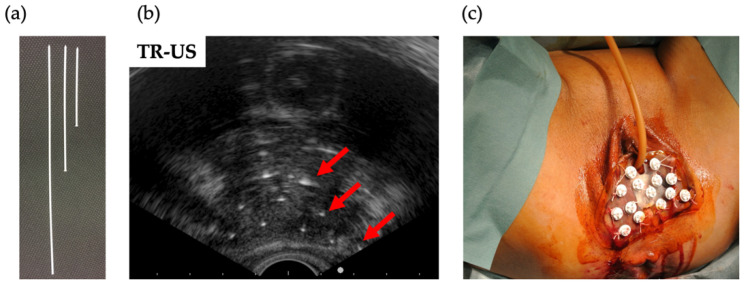
Transrectal ultrasound-guided perineum interstitial needle insertion. (**a**) One-end plastic interstitial catheter. (**b**) Red arrows indicate plastic catheters placed 1.5–2.0 cm apart. (**c**) the perineum after insertion.

The decision to perform ICBT or ISBT was made before initiating EBRT. The indication criteria for ISBT were as follows: major: (1) bladder or rectal invasion, (2) parametrial invasion grossly reaching the pelvic wall observed on MR images, and (3) parametrial invasion with hydronephrosis; minor: (1) bulky tumor ≥ 4 cm in diameter and (2) difficulty in inserting applicator due to cervico-vaginal stenosis. The adaptation of ISBT could be changed when EBRT was more effective than expected. The ambulatory technique designed by Yoshida et al. was adopted at our institution [10,11]. Under spinal anesthesia, interstitial plastic catheters were free-handedly inserted through the perineum guided by transrectal ultrasound (Figure 2). The position of the catheter was checked and, if necessary, adjusted under CT guidance. After catheter insertion, CT and MRI were performed for treatment planning. CT was performed to contour the tumor (high-risk clinical target volume: HR-CTV) and the rectum and bladder (OARs) using the planning system function of CT/MRI registration. ISBT was planned to deliver the D100 (minimum dose covering 100%) of 5–6 Gy per fraction to the HR-CTV. The dose to OARs was aimed at keeping the D2cc (minimum dose delivered to the highest irradiated 2 cm^3^ area) as low as possible (below 5–6 Gy). CT was performed before treatment to determine the position of the interstitial needles. Before every CT, MRI, and brachytherapy, a urinary catheter was placed into the bladder and 100 mL of saline was injected after flattening the bladder, and rectal gas was removed. An ISBT dose of 25–30 Gy in five fractions for three days (two times a day at a 6 h interval) was administered. All patients were treated with iridium-192 HDR remote afterloading systems.

The biologically equivalent dose in 2 Gy fractions (EQD2) was calculated from the combined dose of EBRT and brachytherapy, as previously reported [12]. A value of α/β = 10 was assumed for tumors, and a value of α/β = 3 was assumed for OARs. Dose summation was calculated using only the WPRT dose, and the contribution of the CS dose was not taken into consideration.

### 2.4. Follow-Up

Patients were followed-up and clinically examined by gynecological oncologists every 1–3 months for the first 3 years and every 3–6 months for the following 2 years. Five years after treatment initiation, patients were followed-up every 6–12 months. Disease status and complications were assessed by a medical interview, physical examination, and laboratory tests. Late complications were classified according to the National Cancer Institute Common Terminology Criteria for Adverse Events version 4.0. Pelvic MRI and abdominal CT were performed 3 and 6 months after the initiation of treatment to evaluate the efficacy of the therapy. MRI or CT was performed semi-annually for three years and annually thereafter or earlier in cases of suspected recurrence. 

### 2.5. Statistical Analysis

LC was defined as the interval from the first day of treatment to the date of tumor regrowth and recurrence at the local site. PC was defined the interval from the first day of treatment to the date of tumor regrowth and recurrence within the pelvic field, including the local site. Progression-free survival (PFS) was defined as the interval from the first day of treatment to the date of recurrence at any site. Overall survival (OS) was defined as the interval from the first day of treatment to the date of death. Survival curves were calculated using the Kaplan–Meier method and statistical analyses were conducted using IBM SPSS Statistics version 27 (IBM, Armonk, NY, USA).

## 3. Results

**Figure 3:**
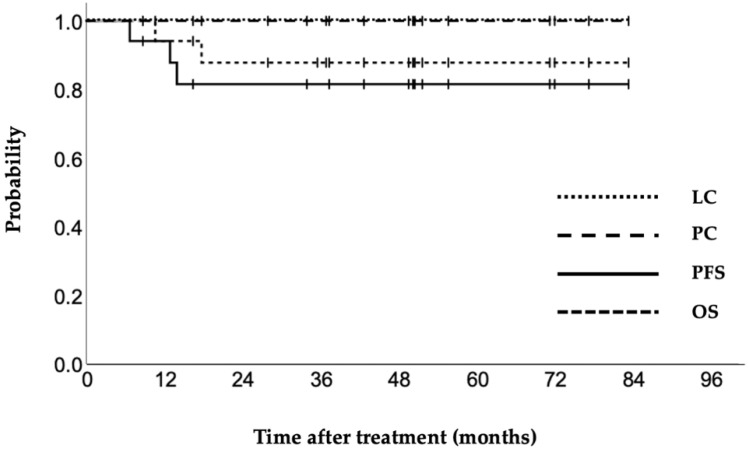
Kaplan–Meier curves for local control (LC), pelvic control (PC), progression-free survival (PFS), and overall survival (OS) of cervical cancer patients treated by CT-MRI-guided multi-catheter interstitial brachytherapy.

The treatment details are presented in Table 2. The median pre-treatment volume was 87.5 cm^3^. Patients received WPRT with a median dose of 30 (range: 30–45) Gy and brachytherapy with a median dose of 30 (range: 25–30) Gy concomitant with a median of 4 cycles of CDDP therapy (range: 0–5 cycles). The median HR-CTV before ISBT was 42.2 cm^3^, and 15 (83.3%) patients had an HR-CTV >30 cm^3^. The median EQD2 for an HR-CTV D90, D95 (minimum dose covering 90% and 95%, respectively), and D100 were 93.1, 90.6, and 79.8 Gy, respectively. The median D2cc for the bladder and rectum were 82.5 and 73.2 Gy, respectively. The four-year LC, PC, PFS, and OS rates were 100%, 100%, 81.6%, and 87.8%, respectively (Figure 3). One patient experienced each of the following grade 3 late complications: rectal bleeding, rectovaginal fistula, and sigmoid perforation (Table 3). No grade 4 or 5 adverse events were observed. The clinical factors of the 18 patients are shown in Table 4. Three patients were not treated with CDDP because of an age over 75 years or renal failure, and four patients were only administered CDDP ≤ 3 times due to ureteral obstruction with renal dysfunction. Four patients with stage IIB cervical cancer underwent ISBT; two cases were due to bulky tumors, and the other two had cervicovaginal stenosis, exhibiting difficulty in inserting the ICBT applicator. Moreover, four patients with bladder invasion required a high dose to the bladder (D2cc >90 Gy); however, at the time of last follow-up, they had not experienced renal or urinary complications. Para-aortic or distant lymph node recurrence occurred in two patients, and upper abdominal dissemination occurred in one. Disease recurrence resulted in the death of these three patients. Finally, none of the patients experienced local or pelvic recurrence during the follow-up period.

## 4. Discussion

CT-MRI-guided multi-catheter ISBT demonstrated impressive clinical outcomes for patients with bulky (≥4 cm) and high-risk, stage IIB-IVB advanced uterine cervical cancer at our institution. Remarkably, the four-year LC and PC rates were both 100%. In contrast, the four-year PFS and OS rates were 81.6% and 87.8%, respectively, due to recurrence outside of the radiation field or distant metastasis. Additionally, three (16.7%) patients experienced grade 3 toxicities after CCRT.

**Figure 4:**
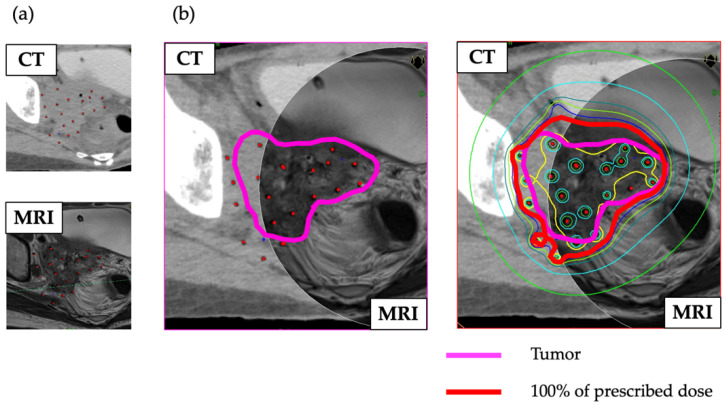
(**a**) CT and MRI of a patient evaluated after implantation. (**b**) Combined CT and MRI of the same patient enabled clear visualization of the tumor contours (pink line). The HR-CTV was mostly covered by the 100% prescribed dose (red line).

The GEC-ESTRO GYN Working Group established in 2000 conducted EMBRACE I, a prospective multicenter study using 3D-IGBT with whole pelvic radiation therapy and weekly concomitant chemotherapy with cisplatin for locally advanced cervical cancer [7]. The RetroEMBRACE study, which was parallel to EMBRACE I, was a multi-institutional retrospective cohort study that demonstrated that IGBT combined with CCRT led to excellent LC (91%), PC (87%), OS (74%), and cancer-specific survival (79%) at three years throughout all stages [13]. Moreover, the results of RetroEMBRACE showed LC rates for stage IB, IIA, IIB, IIIB, and IVA at three years of 98%, 98%, 93%, 79%, and 76%, respectively. A phase II study of CCRT for radiotherapy quality assurance by the Japanese Gynecologic Oncology Group (JGOG1066) showed two-year LC and PC rates of 85% and 73.2%, respectively, using traditional 2D brachytherapy, which was not administered to the tumor without CT-MRI guidance in patients with stage III-IVA cervical cancer < 5 cm in diameter [2]. The study also revealed that the two-year PC rates decreased from 85% to 72% and 54% for tumors with diameters ≤ 50 mm, 51–70 mm, and >70 mm, respectively. These findings suggested that tumors ≥ 5 cm in diameter have poorer outcomes due to insufficient radiation dose coverage of the targeted tumor [14]. In the 2000s, it was reported that MRI-IC/ISBT had better clinical outcomes than ICBT alone for bulky or extensive cervical cancers [3,4]. However, ICBT is occasionally unsuitable for patients with complications, such as cervical atresia and vaginal stenosis. Thus, classical ISBT with metal catheters was used to overcome the problems associated with ICBT. Although MRI is superior to CT for identifying cervical carcinoma extension and delineating the anatomy [15,16], the treatment plan is usually generated with CT imaging because use of a metal catheter is not suitable for MRI. Moreover, classical ISBT is performed in a limited number of institutions owing to the difficulty of its procedure [17]. However, a new ambulatory implant technique consisting of a removable template, flexible plastic catheter, and button stopper has recently been developed [10,11]. This novel technique could not only alleviate patients’ pain during ISBT but also enable clinicians to make treatment plans based on MRI findings after catheter insertion. In addition to this ambulatory technique, the integration of CT and MRI findings for creating treatment plans with ISBT was conceived at our institution. The fused CT and MRI findings improve the localization and visualization of target lesions [18,19], resulting in precise prescription of a sufficient dose for tumors and reduction in bladder and rectal complications (Figure 4). 

The prescribed dose for HR-CTV is a crucial marker for local control of cervical cancer [16]. A previous report showed that cutoff values of >67 and >87 Gy for HR-CTV D100 and HR-CTV D90, respectively, significantly reduced the local recurrence rate, demonstrating a recurrence rate of only 4.4% for an HR-CTV D90 ≥ 87 Gy, compared with 20.5% for doses <87 Gy [20]. Tiwari et al. also concluded that an HR-CTV >85 Gy was a predictive marker for disease-free survival, which was in accordance with the results of the retroEMBRACE study [21,22]. In terms of tumor volume before brachytherapy, the retroEMBRACE study reported three-year LC rates of 93% and 86% for HR-CTVs of 30 cm^3^ and 70 cm^3^, respectively [22]. Furthermore, a systematic review of perineal-based ISBT in cervical cancer patients demonstrated that a lower total EQD2 was linked to inferior LC [23]. These results suggest that LC was correlated with HR-CTV and the dose delivered to the tumor. Moreover, Yoshida et al. reported favorable outcomes after treatment with ISBT for cervical cancer [24]. Patients with a median HR-CTV of 31.8 (range, 10.4–83.2) cm^3^ prior to ISBT were treated with an HR-CTV D90 of 81.9 Gy. The 3-year LC rates were 100%, 95%, and 83% for patients with T2, T3, and T4, and 91%, 90%, and 100% for tumor diameters ≤ 50 mm, 51–70 mm, and >70 mm, respectively [24]. The present study demonstrated an HR-CTV D90 and D100 of 93.1 and 80.0 Gy, respectively, suggesting that 3D-image-guided multi-catheter ISBT could provide a sufficient dose to the tumor. Additionally, the present study included five (27.8%) patients diagnosed with T4a and nine (50.0%) with T3b cervical cancer. Moreover, 15 (83.3%) patients had large tumors (HR-CTV ≥ 30 cm^3^), including four (22.2%) patients with an HR-CTV ≥ 70 cm^3^ before ISBT. Thus, the present study included many cases that were expected to have a very poor prognosis. Additionally, it is noteworthy that none of the patients experienced local or pelvic recurrence during the follow-up period (48.4 months), although generally more than 50% of patients with T3b or T4a cancer experience recurrence within 2 year [25]. These results indicate that 3D-image-guided multi-catheter ISBT achieved remarkable prognostic outcomes for high-risk, locally advanced cervical cancer patients with poor prognosis. Notably, the dose contribution of CS was not considered in the present study; thus, the HR-CTV D90 was higher than the calculated dose, which could have resulted in the favorable outcomes.

We observed the following late complications (grade 2 or higher): vaginal stenosis in two patients, rectal bleeding in five patients, recto-vaginal fistula in one patient, and sigmoid perforation in one patient. One case each of grade 3 rectal bleeding, rectovaginal fistula, and sigmoid perforation was observed among them. Sigmoid perforation occurred 30 months after ISBT and was treated surgically. The pathological findings revealed ischemic colitis with ulceration due to irradiation. In terms of late complications, the grade 3 late toxicity rate of 16.7% was consistent with previous reports of grade 3 and 4 late toxicity rates of 13.8–16.8% [24,26]. Additionally, we observed no serious procedure-related complications, consistent with the study by Mendez et al., which found procedure-related complications of perineal ISBT to be rare [23].

Georg et al. demonstrated that the D2cc (rectum) is a predictive factor for late rectal toxicity [27,28]. They also reported that the D2cc (rectum) was 75 Gy for patients with grade 2–4 late rectal complications [27]. Meanwhile, Yoshida et al. reported that the D2cc (rectum) in cases of grade 2–4 rectal bleeding was 85 Gy [11]. In the present study, the D2cc (rectum) was 69.0 Gy in cases with grade 1 or no rectal bleeding and 75.5 Gy in cases with grade 2 or 3 rectal bleeding. As previously reported, an increased D2cc (rectum) could be a risk factor for rectal bleeding. However, attention should be given to the fact that the dose of OARs in the present study could be underestimated by excluding the CS dose. In the present study, 5 (27.8%) patients with pT4a tumors had bladder involvement and no rectal invasion. While there were concerns regarding the potential occurrence of vesicovaginal fistulas and perforations due to tumor fragments invading the bladder, no patients with T4a cancer in this study experienced these complications. Unfortunately, the patient with stage IIB cervical cancer experienced a rectovaginal fistula at the site where a tissue sample was taken by colonoscopy 10 months after ISBT. Thus, preventive measures should be taken to reduce complications. Previous reports have demonstrated the efficacy of vaginal gauze packing techniques and bladder-rectum spacer balloons in reducing the D2cc of the rectum and bladder [29,30]. However, the use of these tools was limited to ICBT. Hyaluronate gel injection into the perirectal space and vesicovaginal septum could be an effective option for reducing the rectum and bladder dose in ISBT [31,32], although in patients with T4a tumors, this reduction may inhibit adequate dose administration. Finally, recent reports demonstrated that deep learning neural network algorithms using multimodal imaging had the potential for improving the efficiency of OAR contouring [33,34], suggesting improvement in safety of radiation therapy.

There are several limitations to this study. First, we observed a small number of participants. Although we observed better clinical outcomes than previous studies, our results are less conclusive. Second, the efficacy of 3D-image-guided multi-catheter ISBT in non-SCC cervical cancers was not evaluated; thus, ignoring the pathological bias that radical hysterectomy is the standard treatment for patients with non-SCC cervical cancer at our institution due to their low sensitivity to radiotherapy. Finally, patients with stage IVA cervical cancer with rectal invasion were not included. Therefore, our study does not provide evidence that multi-catheter ISBT is safe and effective for such patients. Further studies are needed to clarify the effect of 3D-image-guided multi-catheter ISBT on high-risk, locally advanced cervical cancer.

## 5. Conclusions

In this study, we demonstrated that 3D-image-guided multi-catheter ISBT for bulky and high-risk, stage IIB-IVB advanced cervical cancer leads to excellent local and pelvic control rates. The incidence of complications did not increase compared to previous reports. In conclusion, 3D-image-guided multi-catheter ISBT could prescribe the appropriate dose to high-risk advanced uterine cervical cancer by delineating the clinical target clearly. This method was demonstrated to be a practical and promising breakthrough to reduce poor clinical outcomes. 

## Figures and Tables

**Table 1:** Patient characteristics (*n* = 18).

Variation		Median (Range) or No. (%)
Age (years)		64 (24–82)
Follow-up period (month)		48.4 (9.1–87.5)
Histopathologidal type	SCC	18 (100.0)
	non-SCC	0 (0)
Tumor diameter (mm)	40–60	10 (55.6)
	>60	8 (44.4)
T stage *^1^	T2b	4 (22.2)
	T3b	9 (50.0)
	T4a	5 (27.8)
N stage *^1^	N0	7 (38.9)
	N1	11 (61.1)
M stage *^1^	M0	16 (88.9)
	MA	2 (11.1)
FIGO stage *^2^	IIB	4 (22.2)
	IIIB	7 (38.9)
	IVA	5 (27.8)
	IVB (MA)	2 (11.1)
Lymph node	negative	7 (38.9)
	positive	11 (61.1)

Abbreviation: MA = metastasis to para-aortic lymph node. *^1^: UICC 7th edition, *^2^: FIGO 2008.

**Table 2:** Treatment details (*n* = 18).

Variation	Median (Range) or No. (%)
Median dose to whole pelvis (Gy)	30 (30–45)
Median dose of brachytherapy (Gy)	30 (25–30)
Treatment time (days)	43 (38–55)
Pre treatment volume (cm^3^)	87.6 (31.4–266.7)
HR-CTV volume (cm^3^)	42.2 (17.5–147.0)
≤30 cm^3^	5 (27.8)
>30 cm^3^	13 (72.2)
Median HR-CTV D90 (Gy)	93.1 (79.6–100.0)
Median HR-CTV D95 (Gy)	90.6 (77.6–94.7)
Median HR-CTV D100 (Gy)	79.8 (70.0–80.0)
Median dose of bladder D2cc (Gy)	82.5 (64.8–129.9)
Median dose of rectum D2cc (Gy)	73.2 (53.8–85.4)
Concurrent chemotherapy (cycle)	4 (0–5)

Abbreviation: CTV = clinical target volume.

**Table 3:** Late complications.

Events	Grade 1	Grade 2	Grade 3	Grade 4
	*n* (%)	*n* (%)	*n* (%)	*n* (%)
Gastrointestinal				
Obstruction	1 (6.7)	0	0	0
Proctitis	2 (11.1)	0	0	0
hemorrhage	5 (27.8)	4 (22.2)	1 (5.6)	0
perforation	-	0	1 (5.6)	0
Renal/urinary				
Cystitis	0	0	0	0
hematuria	0	0	0	0
obstruction	0	0	0	0
Genital				
obstruction	0	2 (11.1)	0	0
vaginal fistula	0	0	1 (5.6)	0
Other				
Edema	4 (22.2)	0	0	0

**Table 4:** Clinical variables of 18 patients.

No.	Age	FIGO	Lymph Node	WPRT (Gy)	CDDP (Times)	Pre- Treatment Volume (cm^3^)	HR-CTV Volume (cm^3^)	HR-CTV D90 (Gy)	HR-CTV D100 (Gy)	Bladder D2cc (Gy)	Rectal D2cc (Gy)	Recurrent	Late Complications Grade ≥ 2
1	24	IIB	positive	30	4	130.2	30	79.6	71.8	70	74	none	VO (grade 2)
2	67	IIB	positive	40	5	84.9	17.5	94.7	80	64.8	56.9	none	
3	70	IIB	negative	30	4	31.4	21.4	82.6	70.9	67.4	68.7	none	RVF (grade 3)
4	82	IIB	positive	40	0	43.6	23.7	92.6	80	77.4	81.3	PALN	RB (grade 3)
5	35	IIIB	negative	30	5	77.8	63.6	84.7	70	75.4	58	none	
6	49	IIIB	positive	30	3	208.4	147	94.7	80	94	85.4	DLN	
7	53	IIIB	positive	30	5	54.2	36.3	88.9	70	70	53.8	none	VO (grade 2)
8	69	IIIB	positive	40	5	190.7	133	96.8	80	82.6	73.8	none	RB (grade 2)
9	71	IIIB	negative	30	1	69.2	84.8	85.7	70	84	70	none	RB (grade 2)
10	72	IIIB	negative	30	5	53.1	33.1	82.6	70	68.7	63.1	none	
11	79	IIIB	negative	30	0	45.7	38.4	93.6	80	68	82.6	DIS	
12	48	IVA	negative	40	5	108.4	41.8	98.2	79.6	102.9	52.6	none	
13	62	IVA	positive	40	5	90.2	62.3	97.8	80	118.8	72.6	none	
14	62	IVA	positive	40	0	266.7	30	95.7	80	103.4	81.3	none	SP (grade 3)
15	68	IVA	negative	40	4	60.1	42.6	100	80	115.2	77.4	none	RB (grade 2)
16	77	IVA	positive	40	1	121.2	46.1	97.8	80	129.9	78.7	none	
17	41	IVB	positive	45	5	152	86.2	87.8	76.3	82.4	75.2	none	RB (grade 2)
18	54	IVB	positive	45	2	129	48.9	88.7	76.2	83.7	69.8	none	

Abbreviation: WPRT = whole pelvic external beam radiotherapy; HR-CTV = high-risk clinical target volume; PALN = pala-aortic lymph node; DLN = distant lymph node; DIS = dissemination; VO = vaginal obstruction; RVF = recto-vaginal fistula; RB = rectal bleeding; SP; sigmoid colon perforation.

## Data Availability

The data generated or analyzed during this study are available from the corresponding author on reasonable request. The data are not publicly available due to them containing information that could compromise participant’s privacy.
